# Childhood obesity and comorbidities-related perspective and experience of parents from Black and Asian minority ethnicities in England: a qualitative study

**DOI:** 10.3389/fpubh.2024.1399276

**Published:** 2024-08-05

**Authors:** George Obita, Mark Burns, Lawrence Achilles Nnyanzi, Chia-Hua Kuo, Noël C. Barengo, Ahmad Alkhatib

**Affiliations:** ^1^School of Health and Life Sciences, Teesside University, Middlesbrough, United Kingdom; ^2^James Cook University Hospital, South Tees Hospital Trusts, Middlesbrough, United Kingdom; ^3^Institute of Sport Science, University of Taipei, Taipei, Taiwan; ^4^Herbert Wertheim College of Medicine, Florida International University, Miami, FL, United States; ^5^Escuela Superior de Medicina, Universidad Nacional de Mar del Plata, Mar del Plata, Argentina; ^6^College of Life Sciences, Birmingham City University, Birmingham, United Kingdom

**Keywords:** obesity interventions, barriers and facilitators, NCD morbidity, ethnicity health disparity, qualitative interviews, physical activity, nutrition behaviours, healthcare policy

## Abstract

**Background:**

Preventing childhood obesity and associated comorbidities is often hampered by disproportionate disparity in healthcare provision in minority ethnic populations. This study contextualized factors influencing childhood obesity and related comorbidity from the perspectives and experiences of parents of ethnic minority populations.

**Methods:**

Following ethical approval, families (*n* = 180) from ethnic minority populations in the Northeast of England were contacted through flyers, community social groups and online forum. Of the 180 families contacted, 22 expressed interests, of whom 12 parents were eligible to participate in the study, and one family dropped out due to time constraints. Therefore 11 parents from ethnic minority communities living with at least one child with obesity were interviewed. Each family was separately visited at home and took part in a semi-structured interview based on the study’s qualitative, descriptive phenomenological design. Nine of the families had one child who was diagnosed with an obesity-related comorbidity (non-alcoholic fatty liver disease, musculoskeletal problems or respiratory disorder). Semi-structured interviews were standardized around parents’ perspective and experience on how their children were impacted by obesity and comorbidities, healthcare preventative interventions including lifestyle physical activity and nutrition, and views on tackling obesity impact on their lives. All interviews were analyzed using qualitative thematic analysis.

**Results:**

Parents’ perspectives revealed 11 themes centered around experience of living with a child with obesity, risks, and impact of obesity related Non-Communicable Diseases; and access to support, and barriers unique to minority ethnic groups. Parents revealed social disadvantages, fear of victimization by social services, perceptions on their cultural and religious traditions, and racial stigmatization related to their child’s weight. Parents reported closer bonding with their children to protect them from the untoward consequences of overweight, and little awareness of healthcare obesity prevention programs. Work pressure, lack of time, absence of guidance from professionals were seen as barriers to healthy lifestyle, while support from friends and closer family bond in adopting healthy lifestyle behaviors were facilitators. However, there was little awareness or access to current healthcare obesity preventive offerings.

**Conclusion:**

Minority ethnic communities’ perspective on childhood obesity prevention does not match the healthcare system preventative offerings. Community and family-oriented obesity preventative approaches, especially lifestyle interventions are needed beyond those administered by the primary healthcare system.

## Background

Childhood obesity is a major healthcare concern worldwide, especially in developed countries including the USA and Western Europe (1). The UK ranks 4th highest in the rate of childhood obesity among the 15 countries in Western Europe (2). Disparity in the prevalence and prevention of obesity and associated cardio-metabolic risk is also a major public health concern, especially amongst children (3–5). In England, childhood obesity and overweight prevalence were reported to be 9.7 and 20.2%, respectively, in 2018, but the prevalence of obesity among children residing in most deprived areas was more than double those living in the least deprived areas (6). The highest rate of combined overweight and obesity in England was reported among children from Black, Asian and Minority Ethnic (BAME) communities at 30.8% in children in the reception year (4–5 year olds) and 46% in children in the year six group (10–11 year olds) (7). It is unlikely that such recent disparities are resolved using existing policies, especially post the COVID-19 pandemic which exacerbated obesity comorbidities in BAME communities across the UK (8).

Inequities related to health-related outcomes are presenting a major public health challenge due to its link with reduced life expectancy and quality of life in affected communities (7–12). In the USA, African Americans’ life expectancy was about 6 years lower than their White counterparts in 2020 and are at higher mortality risk from preventable conditions such as obesity and associated comorbidities (9). Ethnic inequities have also been reported in several health surveys conducted in England across all age-groups including children and adolescents (10–13). For example, compared to White children, the odds of obesity were higher among Black Caribbean children, whereas Pakistani children had 40% lower odds. Black African children on the other hand were 40% more likely to be overweight (14). In addition, children from BAME background are more likely to be exposed to an obesogenic environment, especially related to lifestyle compared than their white peers. In a s cross sectional study of the relationship between ethnicity and obesity-related behaviors among school-aged children, Falconer et al. reported that, Black and Asian children were three times more likely to have an obesogenic lifestyle than white children (OR 3.0, 95% CI 2.1–4.2 for Asian children; OR 3.4, 95% CI 2.7–4.3 for black children) (15). Children from BAME background also been shown to have a higher prevalence of type 2 diabetes (T2D) than their White counterparts, 1.42/100,000 vs. 0.1/100,000 and HbA1c difference of 2.1 percentage points (16, 17). Given that BAME communities are the most affected by childhood obesity and associated risks, especially Non-Communicable Diseases (NCDs) and obesogenic lifestyle, there is urgent need for effective interventions among this high-risk group.

Lifestyle behavioral interventions are already established as an effective strategy for preventing obesity and associated comorbidities in both adults and children (18–21). Obesity prevention targeted at family and community levels that starts early in life is likely to be more effective if families become actively involved (22). Recent lifestyle physical activity and nutritional based models have been proposed based on addressing cultural factors and direct intervention approaches based on age and risk stratifications (23). Insight into families’ perspectives and experiences is therefore likely lead to better understanding of the cultural context to design appropriate interventions. For example, culturally adapted nutritional and physical activity assessment design (appropriated settings, questionnaires) may provide better information on lifestyle intervention effectiveness on obesity and related comorbidities, compared with a population wide survey (24). However, our recent systematic review has shown that such contextualized lifestyle interventions are scarce (25). Although limited, a previous questionnaire-based report on parents’ experiences living with a child with a long-term health condition has helped to understand how a child’s illness’ impacts the family’s life, and their responses to the illness that can help professionals develop appropriate prevention, care and support services that meet the child’s and family’s needs (26).

Furthermore, Patel et al. conducted a narrative review on the barriers and facilitators to interventions for T2D prevention in minority ethnic population in the UK, focusing on knowledge and attitudes about T2D; knowledge and attitudes about PA and diet; and barriers to living a healthier lifestyle. This review however was about adulthood T2D and only included studies conducted in the South Asian populations living in the UK (27). Their findings that lack of knowledge about PA and language barriers to a healthy lifestyle exist, though they did not address childhood obesity specific questions. Therefore, extending such knowledge to understand the specific obesity and comorbidities issues among BAME communities from parents’ perspective, is likely to better inform lifestyle obesity and associated NCD prevention and care in those high-risk populations.

Global recommendation by WHO emphasizes interventions targeted at family and communities because of the common behavioral risk factors that start early in the life course (28). In the UK, current local preventative health policies of childhood obesity revolve around primary care approaches of National Health Service (NHS) such as time limited GP centered advice, inadequately resourced referrals pathways based on weight including under-resourced clinics for complications from excess weight (CEWs), and non-specialized healthy weight coaches (HWCs) (29, 30). Intervention approaches aimed toward high-risk populations from minority populations are scarce, despite promising evidence on the effectiveness of culturally relevant support for co-production in preventative health approaches (31).

Capturing parents’ experiences and perspectives on childhood obesity and related comorbidities will potentially provide appropriate qualitative evidence that contributes to designing targeted lifestyle prevention and wider care, management, and support for the high-risk populations. It also provides a snapshot of local experience of minority populations to inform reducing health inequality policies. In the absence of such specific knowledge, this study aimed to capture the perspectives and experience of families from ethnic minorities with a child with obesity and related comorbidity in the Northeast of England. We hypothesized that perspectives and experience of minority ethnicity families do not reflect the support being offered by health authorities.

## Methods

### Design and participants

A descriptive phenomenology design was used to capture parents’ perspectives and experiences in this study. Ethical approval to conduct the study was obtained from Teesside University School of Health and Life Sciences Research Ethics and Governance Committee and a written informed consent was obtained from each participant on the day of the interview. Parents were included in the study if there were aged 18 years and above; were from BAME community; were living in the UK at the time of the interview; were able to effectively communicate in English; and had at least one child with obesity and associated comorbidity. Parents were excluded if they needed a translator for English or unwilling to share their experiences and perceptions in their own words. Comorbidity was defined as a medical or psychological condition that is associated with obesity as the index condition (32). One of the parents (mother or father) of a child with obesity comorbidity was purposively selected to ensure diversity of place of origin and background based on BAME definition in the UK, which refers to non-White ethnic minority (see participant characteristics, Table 1) (33). All participants were located in Middlesbrough city, north Yorkshire County, in the North East of England, UK. A team of experienced researchers: AA, senior research professor of public health, two medical doctors GO (also a DPH postgraduate researcher) and MB pediatric consultant, and (two postgraduate MPH students) were involved in validating the interview process (questions, recordings, transcription, and analyses). Two trained interviewers were also from a BAME background managed all interview meetings and ensured cultural sensitivity when conducting the interviews. All interviews were conducted in the home settings using similar procedures.

**Table 1:** Characteristics of parents and children affected by obesity and related comorbidities.

Characteristics	Number of parents/children
Parents’ characteristics (*n* = 11)	
Age range (years)	31–53
Sex	
Mother	4
Father	7
Region of origin	
Asia	5
Africa	6
Religious background	
Christian	7
Hindu	4
Muslim	0
Others	0
Length of residence in UK	
≥2 years	11
<2 years	0
Relationship status	
Married/living with someone	11
Separated/divorced/widowed	0
Single	0
Range of number of children	1–5
Average number of children	2
Relationship with the child	
Father	7
Mother	4
Carer/guardian/adopted child	0
Affected children’s characteristics (*n* = 11)	
Age range (years)	4–18
Sex	
Male	7
Female	4
Obesity/comorbidity diagnosis	
Self-diagnosed	2
Doctor diagnosed	9
Type of obesity comorbidity	
Non-alcoholic fatty liver disease (NAFLD)	2
Pulmonary disorder/ breathing problems	5
Musculoskeletal	2
Psychological comorbidities	4
Gall stone	1

**Figure 1:**
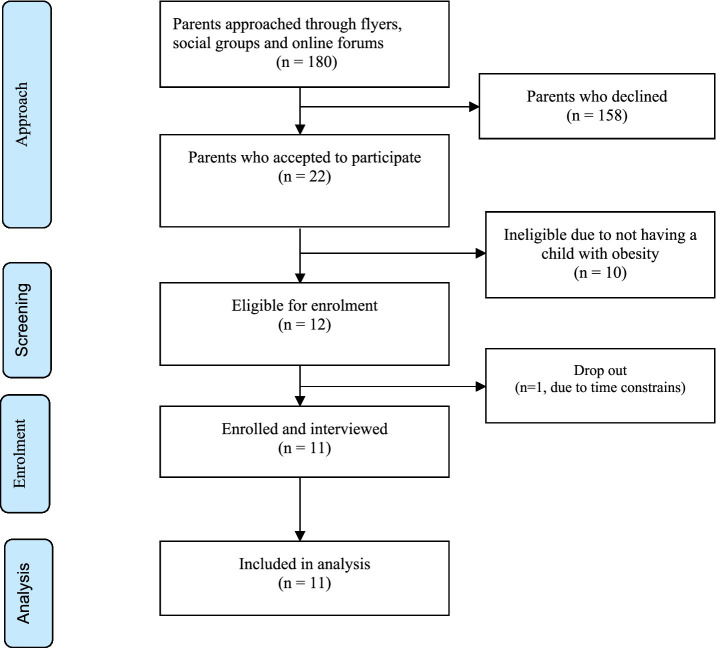
Flowchart diagram for participants selection.

The recruitment strategy involved participants being identified through community social groups, flyers, and online forums (WhatsApp and Facebook groups), which reached a pool of approximately 180 parents from BAME communities living in the Northeast of England. A telephone conversation followed with participants who expressed interest (*n* = 22), who were further screened for eligibility and were provided with a flyer to confirm their willingness to participate in the study. Eligible participants (*n* = 12) received a detailed participant information sheet outlining the purpose, benefits, and procedures of the study, including a copy of consent sheet. A follow up telephone conversation was made to arrange for the interview location and settings with each participant. The final number was 11 participants, with one participant dropping out reporting time constraints. The required sample size (*n* = 11) was calculated based on the model proposed by Fugard and Potts for qualitative studies to achieve a theme prevalence of 0.5, and 5–10 instances, obtaining a sample size similar to previous study designs (34, 35) (Figure 1).

### Procedures and protocol

Written consents were obtained on the day of the interview, followed by a one-to-one in-depth semi-structured Interviews. Interviews were conducted by two trained researchers who were MPH postgraduate students with close supervision by two experienced researchers (AA and GO). All interviews were conducted with one of the parents of a child with obesity comorbidity, in the homes of participants. Each interview lasted approximately 45–60 min. A topic guide with open-ended questions (see Supplementary material), that covered areas directly linked to the aim of the study was used during interviews. The interviewer started by introductions and proceeded to ask participants sensitively and in a conversational manner on how the parent knew their child had obesity and related comorbidity. Subsequent prompts from the interview guide were used to explore perceptions and experiences of parents. There was minimal interruption when parents were describing their experiences. The interview was concluded when the interviewee indicated that they answered the interview questions fully. Participants were given opportunity to raise issues important to them throughout the interview process.

### Data analysis

All the interviews were audio recorded using MP4 audio recorder of iPhone 12 (model number A2403, UK, 2020). Recordings were then transcribed verbatim to ensure that all important information is captured.

Transcripts of the interviews underwent rigorous analysis involving seven stages. In the first stage, GO and AA read and re-read the transcript of the first interview several times to get the gist of the perspectives and experience of parents. During the readings, notes and comments were recorded in the left margin of the page. The second stage GO reread the transcript of the first interview whilst transforming initial notes into codes and themes recorded on the right margin of the page. This was then reviewed by AA. During the third stage, GO related the themes to perspectives on obesity and related illness as a health problem and risk factor; facilitators and barriers to healthy lifestyle to prevent obesity and related illness; experience and impact of living with a child with obesity; and experience of healthcare services and social support while capturing related participants word’s. This process was again reviewed by AA. In the fourth stage, GO and AA individually examined the emerging themes of parents’ perceptions and experiences of obesity/lifestyle in detail, clustering them where appropriate and giving a descriptive name that convey the concepts of the theme, leading to production of a table of themes associated with the priori topic areas of childhood obesity and related comorbidities. Discrepancies were resolved through discussions. In the fifth stage the remaining transcripts of interviews were analyzed in a similar manner to the first transcript. Upon analysis of all the interview transcripts a master list of superordinate and sub-themes of perceptions and experiences of parents of children with obesity comorbidity were produced in the sixth stage of the analysis by GO. The seventh and final stage comprised of writing the report on the findings of the analysis and validating the main themes, capturing parent’s experience and perspective on childhood obesity and related comorbidities by GO and AA.

Microsoft Word (Mac version 16.30, 2019) was used to help organize analysis of data through all the stages and create a trail of decisions and themes identified.

## Results

### Participant characteristics

A total of 11 parents took part in the interviews. Four were mothers and seven fathers, all of whom were employed. Six participants were of African origin and five were of Asian origin (Tables 1, 2). The age range of parents was 31–53 years with a median of 41 years. On average, each parent had 2 children. All families were biological parents of the children who resided in the UK (Northeast of England), for more than 2 years and have used the NHS system as their point of primary care. All children were in the obesity range (Body Mass Index (BMI) > 30 or above 95th percentile for age and sex) and nine children were with diagnosed comorbidity, four of whom had more than one comorbidity. The reported age range of affected children was 4–18 years.

**Table 2:** Dyads details of the parent interviewed and the child with obesity comorbidity.

Dyad number	Dyad details
1	-Mother: South Asian, employed, four years in the UK. -Daughter: 18 years old, with obesity, NAFLD and anxiety.
2	-Father: South Asian, employed, seven years in the UK. -Son: Nine years old, with obesity, sleep apnoea and anxiety.
3	-Father: South Asian, employed, five years in the UK. -Daughter: Eight years old, with obesity and breathing problem.
4	-Father: South Asian, employed, three years in the UK. -Son: 14 years old, with obesity, joint problems, breathing problem and gall stones.
5	-Mother: South Asian, employed, five years in the UK. -Son: 11 years old, with obesity, NAFLD, breathing problem and anxiety.
6	-Father: African, employed, seven years in the UK. -Son: Six years old, with obesity and sleep apnoea.
7	-Mother: African, employed, lived in the UK for 10 years. -Daughter: 15 years, with obesity, joint problems, sleep apnoea and anxiety.
8	-Father: African, employed, five years in the UK. -Son: 11 years old, with obesity and sleep apnoea.
9	-Mother: African, employed, three years in the UK. -Daughter: Four years old, with obesity, joint pains and sleep apnoea.
10	-Father: African, employed, six years in the UK. -Son: Four years old, with obesity and breathing problems.
11	-Father: African, employed, five years in the UK. -Son: six years old, with obesity and breathing problems.

### Main themes

A total 11 main themes resulted from the in-depth interviews, that have been categorised under the different priori topic areas as shown below in Table 3.

**Table 3:** Topics explored with the parents and the main themes being identified.

Topic area	Main theme
Parents perspectives on obesity and related illness as a problem and risk factors	*Acknowledging excessive weight as problem*
	*Childs excessive weight an expected occurrence*
Facilitators and barriers to healthy lifestyle to prevent obesity and related illness	*Family working together and supportive of the child as facilitator of healthy lifestyle.*
	*Lack of time to supervise diet and physical activities*
	*Lack of knowledge and fear of victimization*
	*Unconducive environment and fear of safety of the child*
Experience and impact of living with a child with obesity	*Emotional burden*
	*Stigma and bullying, including racial stigma*
	*Improved family bond*
Experience of healthcare services and social support	*Support from family and friends*
	*Inadequate support from services*

### Parents perspectives on obesity and related illness as a problem and risk factors

How the parents recognized that a child had overweight/obesity and related illness was explored. During interviews parents described how they recognized that their child had a problem related to weight and what they did about it. They described their own observation as well as those of health profession. Two themes were developed as results of the interviews with the parents, namely “acknowledging excessive weight as problem” and “seeing overweight as an expected occurrence.”

#### Acknowledging excessive weight as problem

Most of the parents (*n* = 9) acknowledged excessive weight gain as a problem and described their own observation of symptoms as well as assessment by health care professionals.

For example, an Asian father of a 9-year old son stated: … *“I only noticed he started putting on excessive weight, having difficulties sleeping at night, mostly snoring. So, as a parent we were more concerned right? So, we try to access the health centre, see a doctor and then the doctor did some examination.”*

An Asian mother of a 18 year old girls stated: *…“Yeah, when she was in her teenage, when, shots start of her teenage itself, we find that she has more weight than her friends and having irregular periods, and so because of that reason we just consult the doctor and by doing scanning and all we find that she is having cyst on her ovaries and no, yeah, she’s on medication for the polycystic ovarian disease.”*

In addition, most the parents (*n* = 8) described lifestyle factors such as eating unhealthy diet, lack of physical activities and indoor sedentary lifestyle as the main reasons/risk factors for excessive weight gain in their children. For example, some parents said:

- *An Asian father of a 8-year-old girls said …“I will say the major reason for her condition is eating too much of junk food. She loves burgers, pizzas very much. Even we did not educate her regarding her food habits at that time. We never thought she will face a problem like this in future.”*

The Asian mother of the 18-year-old girl also said: *…“The main factor is the physical inactivity, as she is very lazy to stand up and go to toilet” “Her main hobby is staying in bed and using her mobile phone, also sleeps whenever she is in home in daytime and will be awake at night and use mobile phones” …*

This suggests parent clearly recognized obesity and associated comorbidity as a serious health problem and attribute unhealthy lifestyle such as unhealthy diet, lack of physical activities and sedentary lifestyle as contributing factors.

#### Childs excessive weight was expected occurrence

Some parents (*n* = 3), on the contrary, saw weight gain in their children because of what the parents passed on to the children and felt that it was inevitable. Excessive weight was viewed as a consequent of parents’ genes or what the mother consumed during pregnancy. For example, some parents said the following:

An Asian father of a 9-year-old boy said: …*“my father and my wife’s father side, they had problem being excess in weight, Yes, my father had type 2 diabetes”…*

An African father of an 11-year-old boy said: *… “I think it might be genetical, but yes, nutrition can be a cause for that because during pregnancy, my wife used to eat a lot and different kinds of foods for the baby health” …*

An African father of a 4-year-old boy said: *… “He did not have any predisposition apart from may be taking my gene. The mother is with the normal weight, but myself I’m a bit overweight, so that might maybe have a risk factor on child.” “I was obese when I was a young child, so I think that’s the reason why” …*

### Barriers and facilitators to healthy lifestyle to prevent obesity and related illness

Four themes were developed under this area of exploration from what parents expressed and reflected on. These include lack of time to supervise diet and physical activities; lack of knowledge; and unconducive environment and fear of safety of the child as the main barriers. Little was expressed of facilitators of healthy lifestyle apart from support from friends and family.

### Barriers

#### Lack of time to supervise diet and physical activities or prepare healthy food options

Lack of supervision by parents (*n* = 8) due to their busy schedule was identified as one of the barriers to their children living a healthy lifestyle.

For example, an African mother of a 4-year-old son said: *… “Free time too because yeah you must watch your child. It’s you have to like even when they go out for any physical activity or any sports or whatever. You have to be there watching each other. Most times I do not have that time.”…*

Another parent, an African mother of a 15-year-old girl said: *… “lack of time to prepare healthy food due to work pressure”….*

Lack of time therefore hindered the ability of parents to supervise their children physical activity or participate in physical activities with their children.

#### Lack of knowledge

Some parents (*n* = 4) expressed what can be considered the lack of knowledge of the danger of poor dietary habits as result of traditional/cultural belief. Further, they expressed fears of social services if a child looks thin, so they have to over feed the child to look well nourished. Here is what some of the parents said:

An African father of an 11-year-old girl said *… “over feeding because of love, because of the euphoria of having a child. And then we give them a lot of things to eat, the easily available…” “Yeah, and then not realising that you have to start watching these things sometime then secondly then” “Perception that will be penalised by social services if the child looks undernourished”…. “People may think we are not taking good care:….*

An African mother of a 4-year-old boy said *….“Like in Africa, most times you would measure, sometimes not always. You’d measure like if. Like a child, if the child is plump and then you would say oh this child is well fed, you know you’d see the parents are OK, they are doing well because you can see that the child is well fed, is not skinny”….*

#### Unconducive environment and fear of safety of the child

Barriers to physical activities included fear of safety of the child playing outside. Some parents (*n* = 4) expressed that due to fear of safety of their children they often do not allow them to go and play outdoors.

For example, an Asian father of a 8-year-old girl said *… “Fear for safety, so keep the child indoors”….*

A parent identified change of the environment from their country of origin to the UK as a barrier to children living a healthy lifestyle and therefore a factor in their excessive weight gain. The parent reflected on experiences growing up in country of origin, in contrast to how they live in the UK. The parent recalled children having the freedom to play around and eat organic and natural food in their country of origin whereas in the UK they mostly consume unhealthy junk food as a convenient option and do not play freely outdoors unsupervised.

The African mother of a 4-year-old girl said: *… “Basically, I think it’s about the nutrition and the lifestyle. We moved to the UK like two years ago, I’m from Africa. She had the normal diet like and played around like other children would do in school and everything. But when we moved here, we were not getting exactly like the food that we are used to and we needed to make things like fast meals that they can easily have and get to school”…*

### Facilitator

#### Family working together and supportive of the child activities

Although most parent did not often discuss specific facilitators of healthy lifestyle behaviors, the most mentioned (*n* = 6) factor was support from the family and friends. Here is what some of the parents said:

For example, an African father of a 6-year-old boy said: *… “and that suits your child and try as much as possible to give him other supports for the most important thing also is that each family have their popularity and they probably would develop things that works for them. For me this works for me. Love care support and communication for the family”….*

Another parent, an Asian father of a 14-year-old girl said: *…“We included outdoor activities in our lifestyle especially morning walk, leisure time at park and do yoga”….*

### Experience and impact of living with a child with obesity

#### Emotional burden

During the interviews, the emotional burden of having a child with obesity was expressed was expressed by more parents (*n* = 9). The most common emotion expressed was worry about the child’s future. Conversation with participants demonstrated the impact of this emotional feeling.

For example, an Asian mother of a 18-year-old girls said *… “I was very worried about her, and I felt so bad”….*

An Asian father of a 9-year-old boy said *…“obviously I’m very concerned when I consider my son’s life ahead, what if this obesity causes any other health problems to him in the future” …*

an Asian father of a 8-year-old girl said *… “Initially we were very worried about her condition, whether it’ll go away by time or not, is there any complications that may occur along with obesity in my daughter. But our doctor gave us a clear explanation and routes to change this”…*

Some parents spoke about their child’s weight problems with a feeling of guilt because they did not do enough to prevent it.

For example, an Asian father of a 8-year-old girl said *… “Honestly, I’ve been blaming myself not to educate my daughter about her food habits. But no one cannot do anything about that now” …*

They also expressed how hard it was to control the eating habits of their children and the feeling of loss of control.

For example, an African father of a 4-year-old boy said: *… “They see it as a negative thing, but I do not have control over it. To some extent because sometimes you just go into the fridge and then he picks up anything that he wants so he can. It’s very difficult to control, I mean his food intake, but I know it’s not all that good”…*

Two parents (*n* = 2) expressed no worries as they felt things will resolve themselves as the child grows up.

For example, one of them, an African father of 11-year-old boy said *… “Not worried. “Obesity is not something that we will have to worry so much about because with her behavioural changes and then making inputs to ensure that the child eats well and then play well, we are hoping that we are optimistic that is it enough. By time child grows, he might even outgrow it. Say the child is he’s coping with his peers”…*

#### Stigma and bullying

Stigma and bullying of the affected child, especially within the school environment, was a common feature described by participants (*n* = 6). This stigmatization and bullying often left the children withdrawn, shy and fearful. Some parents said the following during interviews:

An African father of a 7-year-old boy said: *… “Stigma from school because unable to participate in some activities, the child feels bad from the stigma”…*

An Asian father of a 9-year-old boy said: *… “I think in terms of his experience, I think most of the experiences had towards his condition comes from his parents, mostly in school, where he he’s unable to participate in certain kind of sports and people tend to point fingers”…*

An Asian father of a 8-year-old girl said: *… “And all but I know the fact the issue she might have. It’s the issue with students following her in school because most time she comes back I know most times the other brother they you know get into a fight with other students because they tried to bully her sister and all that”…*

An African mother of a 15-year-old girl said: *… “I notice most times these days as she is growing up, she used to be a very happy child. You know, growing up, but these days she looks more withdrawn and most times when they have like social activities in school outside going to school classes. I know that when they have like social activities, she oftentimes does not want to like get involved when they have like performances, they need to draw on stage, even though she knows she can do better. But mostly she does not want to come open in the public glare”…*

#### Improved family bond and healthy lifestyle behaviors

Two participants (*n* = 2) described improved bond and support within the family to psychologically support the unwell child.

An African father of a 11-year-old boy said: *… “There were significant changes in the diet in the family and fixed a time for physical activities like work out and gym. The screen time was also reduced”…*

An Asian father of a 14-year-old boy said: *… “Not being an easy one, having to go in and out of the house. Well aside that my wife has been very, very supportive and be supportive of our son”…*

### Experience of healthcare services and social support

#### Support from family and friends

Participants described experiences of social support was limited to friends and family, where they received support. For example, one participant said that besides her friends and neighbors there was no support from the government. They expressed supportive experiences that varied with type of support and source of support. Common source of support was family and friends.

#### Inadequate support from services

None of the participant described any exposure, knowledge, or access to public health lifestyle program/weight management program. Most of the participants described support they received from their GP/doctors for clinical care and one participant expressed support from social workers for counseling for mental wellbeing. Parents said:

An African father of a 4-year-old boy said: *… “No government supports and there’s nothing”…*

An African father of a 4-year-old boy said: *… “Doctor offered support but because of lack time, have not accessed it”…*

An African father of a 4-year-old boy said: *… “Support and medication from the doctor”…*

## Discussion

This study explored the perspectives and experiences of parents from minority ethnic populations in the Northeast of England on having a child with obesity and related commodities, which included the impact of living while caring for a child with obesity comorbidities, experience of healthcare services and social support, and perceived barriers to lifestyle intervention. The main finding of this study is that most parents understood obesity and related comorbidities are serious health problems that are likely to impact their children negatively in the future. They were also knowledgeable of obesity risk factors, especially lifestyle factors of unhealthy diet, physical inactivity, and sedentary behavior as the main contributors. Despite this awareness, there was little perceived healthcare system preventative support for those communities. Parents reported no access to public health weight management programs, except for limited GP/doctors contact for clinical care, and social workers for psychological support. Instead, parents identified several barriers to obesity prevention, including an unconducive environment, time pressures, fear of safety of their child, lack of information and fear of victimization and cultural perceptions. The latter barriers were combined with parents’ experience of emotional burden and dealing with weight-based racial stigmatization and bullying of their children. Facilitators identified through family’s experience included, cultural social norms and pressure, closer bonding with family relatives and friends for support related to the affected child and practical advice. The study’s insight into parents’ perspectives and experience of minority ethnic communities shows a dichotomy in the perceived healthcare support system and the need to better integrate community’ perspectives in obesity healthcare services and designing effective lifestyle interventions. Policy implications should focus on matching both prevention services and community health provision with the health needs and experiences of communities from minority ethnicities with high obesity and comorbidity risks.

**Figure 2:**
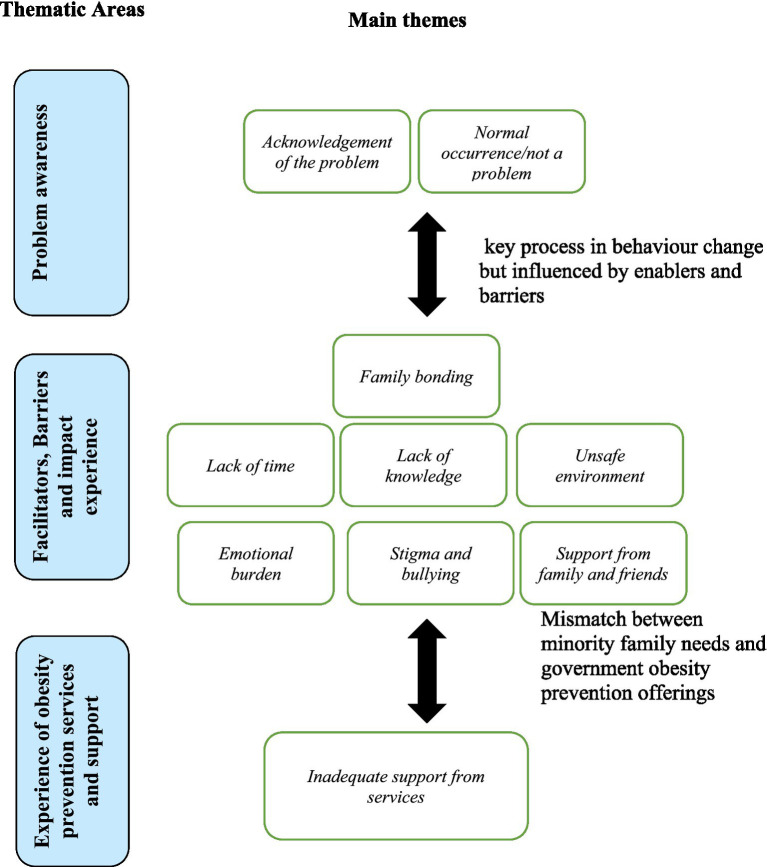
Main findings on the experience and perception of parents of children with obesity comorbidities from minority ethnic communities in England.

Most parents interviewed in the current study had already noticed that their child might have health problems related to weight and sought help from their doctor. Health care providers carried out assessments and informed parents of their child’s overweight/obesity status, nine of whom were diagnosed with a specific obesity related comorbidity, including Non-alcoholic fatty liver disease, pulmonary problem, and psychological problems. This played important influence on parents’ perception of their child’s overweight/obesity status as serious health problem that may affect them negatively in future. Previous studies have also reported that assessment and discussions with healthcare professionals, enables parents to acknowledge that their children’s overweight status might be a health problem (36, 37). The acknowledgement by parents from BAME community that childhood obesity and associated comorbidities are serious health problems is good first step in recognizing the need for lifestyle interventions. However, parents also described barriers to healthy lifestyle related to lack of time to supervise children’s activities, knowledge gap, unconducive environment and fear for child’ safety, corroborating findings from previous studies on ethnicity-specific influences on diet and physical activity behaviors (38–42). Unfortunately, there was no evidence that this group of BAME families and children had accessed any NHS preventative schemes on childhood obesity including the NHS tier-based weight management system, involving referrals for complications from excess weight (e.g., CEWs clinics) to provide intensive lifestyle interventions. However, such clinics are overwhelmed with referrals, which has led to inability to handle existing referrals, let alone to reach BAME populations (43). Other signposting NHS services such as HWCs and National Child Measurement Program have provided little evidence on effectively reaching BAME communities (35, 44, 45). The mismatch between such healthcare provision in preventing obesity and BAME community reach in the present findings (Figure 2), which highlights the need for a more personalized interventions, which engages the community in the design and application of preventing obesity and associated lifestyle diseases (23, 25).

Although no country has succeeded in lowering the prevalence of obesity at national level (46), there are regional accomplishments in lowering childhood obesity prevalence through effective local policies and strategies. For example, New York City saw a decline in obesity prevalence from 21.9 to 20.7%, (5% relative decline) among school children (grades K-8) between 2006 and 2007 and 2010–2011 following implementation of a targeted policy at reducing ethnic, socioeconomic and disability disparities and working in partnership with schools, communities, businesses and various stakeholders (47). However, this decrease was smaller among Black (1.9%) and Hispanic (3.4%) children than among Asian/Pacific Islander (7.6%) and white (12.5%) children, suggesting that this success was primarily driven by White children and less so among minority Black, Hispanic and Asian/Pacific Islander children (47).

A similar policy implementation in Massachusetts (Somerville city) led to reduction of the prevalence of overweight/ obesity in children, BMI z-score decreased by −0.1005 (*p* = 0.001, 95% confidence interval, −0.1151 to −0.0859) compared with children in the control communities over a 3 year period (48). The same intervention positively affected parents by reducing their BMIs by 0.411 points (95% confidence interval = −0.725, −0.097) relative to control parents (49). Such evidence supports that local childhood obesity prevention policies which engages minority ethnic communities (children, parents, teachers, school food service providers, city departments, policy makers, healthcare providers, before-and after-school programs, restaurants, and the media) can successfully address inequalities in childhood obesity prevention. In the absence of a UK childhood obesity prevention policy specifically targeted at BAME communities, the present findings on the experience of healthcare services and social support in a UK context provides an important snapshot toward policy forming in the UK healthcare system.

We recently demonstrated that lifestyle interventions are effective in ethnic minority groups mostly when contextualized and tailored to their cultural norms. Our recent systematic review analyzed over 26,000 children with obesity and related comorbidities and showed that supervised and combined PA and nutrition interventions with low attrition rate are able to reverse obesity and related comorbidities in minority ethnic population (25). For example, Dos Santos et al. in an eight-week randomized intervention trial demonstrated that combined nutrition and physical activity intervention that included improvement in family functioning was effective in reducing BMI (*p* = 0.012) and waist circumference (*p* = 0.001) among Hispanic children (50). Further evidence from US populations showed that culturally adapted interventions improves relevance, feasibility and effectiveness of obesity intervention among ethnic minorities population (51). In another study that used overweight/obese predominantly African Americans and Hispanic American mothers as change agents to improve food choices, and physical activity in their 1-to-3-Year-old children, culturally adapted intervention reduced mothers mean BMI from 34.9 to 33.9 kg/m^2^ by week 8 (*p* < 0.001) and sustained at week 24. For those children, normal growth pattern and height (*p* < 0.001) and weight (*p* < 0.001) for age were sustained during the study (52). Similarly, several RCTs and pre-post quasi-experimental studies have demonstrated that lifestyle interventions are effective in reducing adiposity as well as metabolic comorbidities of childhood obesity such as T2D, hypertension and cardiovascular disease among minority ethnic population when implemented under controlled condition such as guided physical activity, guided nutritional education and environmental changes (50, 53–61). Lifestyle intervention among BAME community in the UK are scarce, but overall assessed evidence from intervention studies around the world in HICs suggests that, if appropriately designed and implemented, lifestyle intervention can be effective in reducing overweight/obesity and metabolic risk factors among children from minority ethnic population (25).

Given that most of children whose parents were interviewed were of school age, policies that promote multilevel and multicomponent approach with family involvement are feasible at the local settings including schools and community. A recent critical review of effectiveness of such policies and strategies for childhood obesity prevention through school based, family and community involvement showed that strategies that integrates school policies of availability of healthy food and beverage choices and limiting unhealthy snacks; encouraging teachers to be active role-models; physical education classes to; and active parental involvement assignments, meetings, and home environment improvement were effective in preventing childhood obesity. Whereas strategies that did not involve policy and environment changes but focused on educational sessions were less effective (62). In this study some parents discussed lack of availability of cultural food options but instead easily availability of fast unhealthy fast food. The understanding of food access, available food options, role of school food and cultural foods available in the home is important for understanding of the dietary habits of children and designing appropriate strategies to address unhealthy nutrition (63). Therefore, health policies should consider the feasibility of implementation of obesity interventions at the local level among minority ethnic communities for it to be effective at population level.

Barriers to healthy lifestyle behavior as described by participants of the present study, included lack of time, unconducive environment, stigma, or lack of effective referral/signposting to appropriate services. This is contrary to the current National Institute for Health and Care Excellence (NICE) guidelines, which recommends tailored lifestyle advice by healthcare services, based on barriers among BAME populations (64). It is therefore unknown how effectively such recommendation is being followed. It is also noted that some parents in this study perceived that their child’s obesity as primarily due to genetic and biological factors, rather than acknowledging the complex interaction between lifestyle and biological factors as contributory to the excessive weight and related problems in their children. This could be considered an element of passive denial behavior that may prevent them accessing appropriate services (65). Therefore, additionally to providing an appropriate access to healthcare services, it is important to continue engaging minority ethnic populations in the design and implementation of appropriate obesity prevention programs to actively increase awareness of the common risk factors experienced by all people, regardless of their culture or ethnic background. This suggestion applies to health services across the UK as our findings corroborate a synthesis of 14 (5 conducted in Scotland and 9 in England) qualitative studies on barriers to physical activity (PA) among adult BAME communities in the UK that found the main barriers to be personal barriers such lack of time and work pressures; limited access to PA due to external factors such as distance to facilities, fear of safety and cost of access to PA; and perceptions and cultural expectations (66).

Parents involved in this study experienced social support from friends, family, and neighbors as the main facilitator of healthy lifestyle. They specifically described support for healthy lifestyle behaviors such as exercise, healthy eating choices, and emotional support in terms of being available to talk and advise. This is consistent with Verheijden et al. report demonstrating a stronger correlation between social support and health outcomes among Black ethnic communities compared with their White ethnic counterparts (67). Similarly, Christakis and Fowler (2007) examined repeated data on 12,067 participants of mixed ethnicities and found and association between obesity and social network in those communities (68). Therefore, supporting positive social environment is an important component in childhood obesity and related comorbidity prevention effort among children from BAME communities and require attention in the design of interventions, especially lifestyle prevention of obesity.

Families additionally experienced negative social consequences due weight-based stigmatization and bullying of their children, commonly in the school environment. They described their child being less socially accepted among their peers, which in turn resulted in psychological problems such as anxiety and social withdrawal. Other studies have similarly reported on weight-based stigmatization among children of all ethnicities, such as that stigmatized children with obesity often experience bullying, teasing, poor self-esteem, psychological disorders, poor school performance, and poor social interactions (69–71). However, weight base stigmatization in children from BAME communities is often associated with additional racial stigmatization and discrimination (72), which has been a themed concern within participants’ experience in this study (Table 3). There seems to be a problem of rising weight-based stigma proportionately to the increased prevalence of obesity amongst the wider populations (73). However, the racial and ethnic dimension of obesity stigma should also be addressed in the context of childhood obesity prevention strategies.

Parents also reported that their children’s participation in school sports and social activities were hampered by racial-related stigma and bullying, which is a known issue reported amongst overweight children (74). Several stigma coping strategies have been suggested including changing the stigmatizing condition through losing weight, and taking pride in the condition and mobilizing social action to prevent discrimination (75). There is no local evidence on whether either approach would work in BAME communities in England. However, our recent reviews and meta-analysis suggest that a more contextualized intensive lifestyle intervention approach to be effective in childhood obesity prevention among ethnic minority populations (25).

### Implication for practice and policy

The current study identified contextual factors, the lived experience that impact on obesity risk and lifestyle behavioral change among families from minority ethnic communities with a child living with obesity and related comorbidities. In particular, the role of social support from family, friends and neighbors needs to be identified and clearly articulated within interventions that address childhood obesity and related NCDs among BAME communities.

The findings also suggest focusing on cultural contextual influences when designing and implementing obesity and associated comorbidities prevention strategies and interventions among minority ethnic communities living in HICs. Such approach will reduce the mismatch between reportedly available healthcare provision and the end-user communities who are considered at high risk of developing lifestyle related diseases including obesity and related comorbidities. While coping strategies with reported obesity stigmatization is recommended for widening participation in obesity preventive healthcare initiatives, such approach should also consider the additional racial and ethnic discrimination faced by minority ethnicities.

In the wider context of reducing health inequality, the study recommends empowering the community in order to reduce weight-based discrimination reported in this study and provide better representations of BAME communities in health leadership and decision making regarding their lifestyle choices and health services (76). Recent national policy statements on tackling obesity (healthier choices, food shopping and marketing) (77), did not seem to address barriers expressed by BAME parental working conditions expressed by parents in this study. We recommend local public health stakeholders in the North East of England (e.g., universities, local authority) to develop a more inclusive policies in the wider context of reducing health inequality and obesity prevention including more diverse decision makers, better BAME representation and engagement in decision making. Nonetheless, the wider health equity implementations from this study apply to all HICs populations.

### Limitations

The study’s participants represented a diverse yet small sample of BAME participants with higher education attainment within the Northeast of England, which helps shed the light on childhood obesity prevention issues faced by minority ethnicities living within a typical Western HIC. However, differences in such perspectives between different HICs such as the USA may occur, especially that differences in the discrepant responsiveness to childhood obesity interventions amongst diverse minority ethnic populations have been reported elsewhere (25, 50, 58, 78). As such, larger sample from multi-country studies are required on the perspectives of minority ethnic populations in other HICs in the wider context of obesity prevention strategies (79). The community’s perspective and contextual factors should be considered when designing appropriate childhood obesity and related NCD interventions. While this study reported on parents’ perspective, future studies should incorporate children and adolescents’ perspectives to incorporate their views and experiences, as well as specific differences amongst different ethnicities. Future similar thematic analysis could employ blind coding using qualitative software instead of the time-consuming manual coding performed here. Nonetheless, manual coding was conducted and checked by two experienced public health researchers (AA, GO) and themes identified were checked by all authors.

## Conclusion

This study contextualized factors influencing childhood obesity and related comorbidity from parents’ perspectives of BAME communities in the Northeast of England, which can contribute to reducing disparity in healthcare provision that have disproportionately negative impact on minority ethnic populations living in HICs. Parents’ awareness of childhood obesity health risks, perspective on limited access to weight management services, and weight-related racial stigmatization all demonstrate a mismatch between healthcare obesity prevention provision and high-risk communities from ethnic minority populations.

Contextual factors should be considered when designing appropriate childhood obesity and related NCD interventions. Community contextualized and family-oriented obesity preventative approaches, especially lifestyle interventions are needed beyond those administered by the primary healthcare system. Future studies need to examine the experience and perspectives children.

## Data availability statement

The raw data supporting the conclusions of this article will be made available by the authors, without undue reservation.

## Ethics statement

The studies involving humans were approved by the School of Health and Life Science Ethics Committee at Teesside University. The studies were conducted in accordance with the local legislation and institutional requirements. The participants provided their written informed consent to participate in this study.

## Author contributions

GO: Data curation, Formal analysis, Methodology, Software, Writing – original draft. MB: Validation, Writing – review & editing. C-HK: Validation, Writing – review & editing. LN: Writing – review & editing, Visualization. NB: Writing – review & editing. AA: Writing – review & editing, Conceptualization, Data curation, Formal analysis, Funding acquisition, Investigation, Methodology, Project administration, Resources, Software, Supervision, Validation, Visualization, Writing – original draft.
