# Supplementary material for: Childhood obesity and comorbidities-related perspective and experience of parents from Black and Asian minority ethnicities in England: a qualitative study
Source: Front Public Health. 2024 Aug 5;12:1399276. doi: 10.3389/fpubh.2024.1399276 (PMC11340680; doi:10.3389/fpubh.2024.1399276)
Supplement: Supplementary file 1 [file Data_Sheet_1.docx]

**Perspectives and *experience of families of children with obesity related comorbidities from Black and Minority Ethnic communities***

Student: ***George Obita***

Academic Supervisor and Chief Investigator: ***Prof.*** ***Dr. Ahmad Alkhatib***

The following thematic topics will be covered and form the content of the questions.

a) Perspectives of risk factors of childhood obesity and comorbidities; b) perspective on link between obesity and comorbidities; c) perspective on role of lifestyle (physical activity, nutrition, sedentary life) as risk factors and prevention; d) perspectives on facilitators and barriers to prevention and support e) experience access to services; f) experience of living with a child with obesity comorbidity and impact on family.

Specifically, the topic guide for the interview will include the following:

1. **Introductions**

- Introduce self and purpose of the meeting
- Obtained verbal and written consent
- Explain recording, confidentiality and right to withdraw

1. **About the family**

- Prompts: How many children do you have (birth order)? What are their names, ages? Who is your partner? Participants role and age group

1. **Identifying the child with obesity comorbidity and how it was diagnosed**

- Prompts: Who in the family has problems with excessive weight and related illness? did you know that they have problems with weight? Is he/she taking medications or attending any clinic? What problem was identified? Who made diagnosis of the problem? When did they begin having problems due to weight? When did you first notice this happening? How did you feel about that? And how do you feel now?

1. **Experience of having a child with obesity comorbidity**

- Prompt: What does it mean to you that (child’s name) has the illness? Has this experience been for you? How does it make you feel? How do you think your partner/family feel about it? How do you think (child’s name) feels about it? What do you think other people think about it?

1. **Perspectives on risk perception**

- Prompts: What do you think might have played a role in your child’s illness? Do you think your nutrition, lifestyle or culture/tradition might have had a ‘role’ in how you view (child’s name) illness?

1. **Impact on family**

- Prompts: Do you think (child’s name) illness has impacted on the family in anyway? /In what way? How are things different now? What did things used to be like in your family? How do you feel about your child? What is your relationship like with [child’s name]? How do you interact with them? Do you notice any patterns of behaviour between the two of you? Has it always been like that (since birth)? If someone was looking in from the outside, how do you think they would describe what your family is going through? How would they describe all of the different difficulties the family is going through? And, all of the different personalities?

1. **Access to services**

- Prompts: What support have you received? What services have you had access to? What is your experience of the services? Are there any support you would have liked to receive?

1. **Prevention and support interventions**

- Prompts: What do you think should be done to support you to prevent such illness occurring or to care for the ill person?
